# Percutaneous Retrieval of Dislodged Chemo Port Catheter With Inaccessible Tips by a Simplified Technique

**DOI:** 10.7759/cureus.21692

**Published:** 2022-01-28

**Authors:** Sanjay C Shah, Rajnikant Radadiya, Aman Patel, Subrahmanya Murti Velamakanni, Tejas Patel

**Affiliations:** 1 Interventional Cardiology, Apex Heart Institute, Ahmedabad, IND; 2 Cardiac Anesthesiology, Apex Heart Institute, Ahmedabad, IND; 3 Cardiology, Smt. Nathiba Hargovandas Lakhmichand Municipal Medical College, Ahmedabad, IND; 4 Cardiology, Apex Heart Institute, Ahmedabad, IND

**Keywords:** fluoroscopy, chemotherapy port, foreign body removal, gooseneck snare, percutaneous cardiac intervention

## Abstract

Central venous port catheters (CVPCs) are commonly employed for long-term chemotherapy. One of the rare complications associated with CVPCs is catheter fracture and further embolization of the fragmented segment into the heart. The most common site of embolization is the superior vena cava-right atrium (RA) junction. However, infrequently, the catheter may embolize further distally into the right ventricle (RV) and beyond making the fragmented tips difficult to access directly with a snare. Here, we report a case wherein both the catheter tips were lodged in the RV cavity forming a loop in the RA. This necessitated the use of a modified technique to retrieve the fragment percutaneously.

## Introduction

Every interventionalist should be well versed with the techniques to retrieve foreign materials from the venous and arterial circulation, as well as the right and left heart [1-3]. Central venous port catheters (CVPCs) are commonly employed for the delivery of long-term chemotherapy. One of the rare complications associated with CVPCs is a fracture of the catheter and its embolization into the heart, the incidence of which has been estimated at 0.4-3.1% [4,5]. The most common site of embolization is the superior vena cava (SVC)-right atrium (RA) junction wherein the tips of the dislodged fragment may be easily accessible by standard snares [6]. However, infrequently, the catheter may embolize further distally into the right ventricle (RV) and beyond making the fragmented tips difficult to access directly with a snare. Here, we report a case wherein both the catheter tips were lodged in the RV cavity forming a loop in the RA. This necessitated the use of a modified wire loop and snare method. First, the fragment was repositioned into the inferior vena cava (IVC) and subsequently snared out directly.

## Case presentation

A 51-year-old female, a known case of carcinoma breast on regular chemotherapy, was found to have a resistance in infusing fluid through the CVPC. A subsequent chest X-ray showed a fracture of the CVPC and dislodgement of the fragment into the RA-RV with a loop. The patient was referred to our center for retrieval of the fragment.

**Figure 1:**
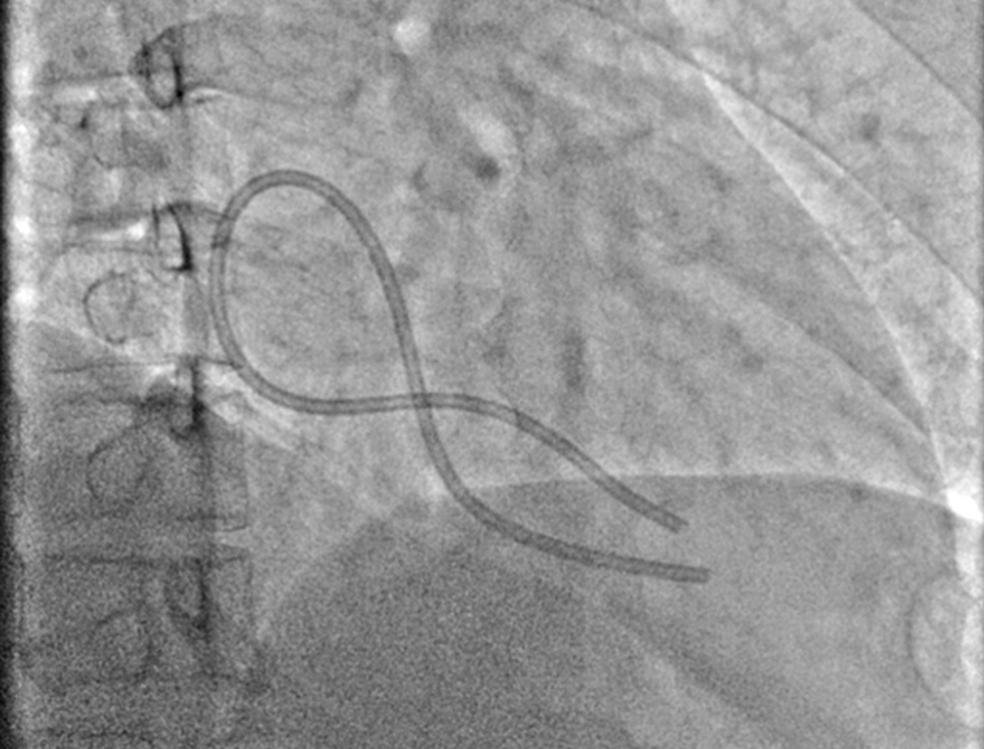
Showing the dislodged chemo port catheter looped in RA with tips in RV on fluoroscopy. RA: right atrium; RV: right ventricle

On fluoroscopy, the CVPC was fractured with a dislodged fragment. Both the tips of the fragment were in the RV forming a loop in the right atrium (Figure 1). Right femoral venous access was taken with a 9 Fr Radifocus Introducer-II (Terumo Corporation, Tokyo, Japan) sheath. A 0.035 inch, 260 cm wire (Cordis Corporation, Santa Clara, CA, USA) was introduced into the RA. A 5 Fr 3.5 Judkins Right (JR) catheter (Cordis Corporation, Santa Clara, CA, USA) was passed over the wire.

**Figure 2:**
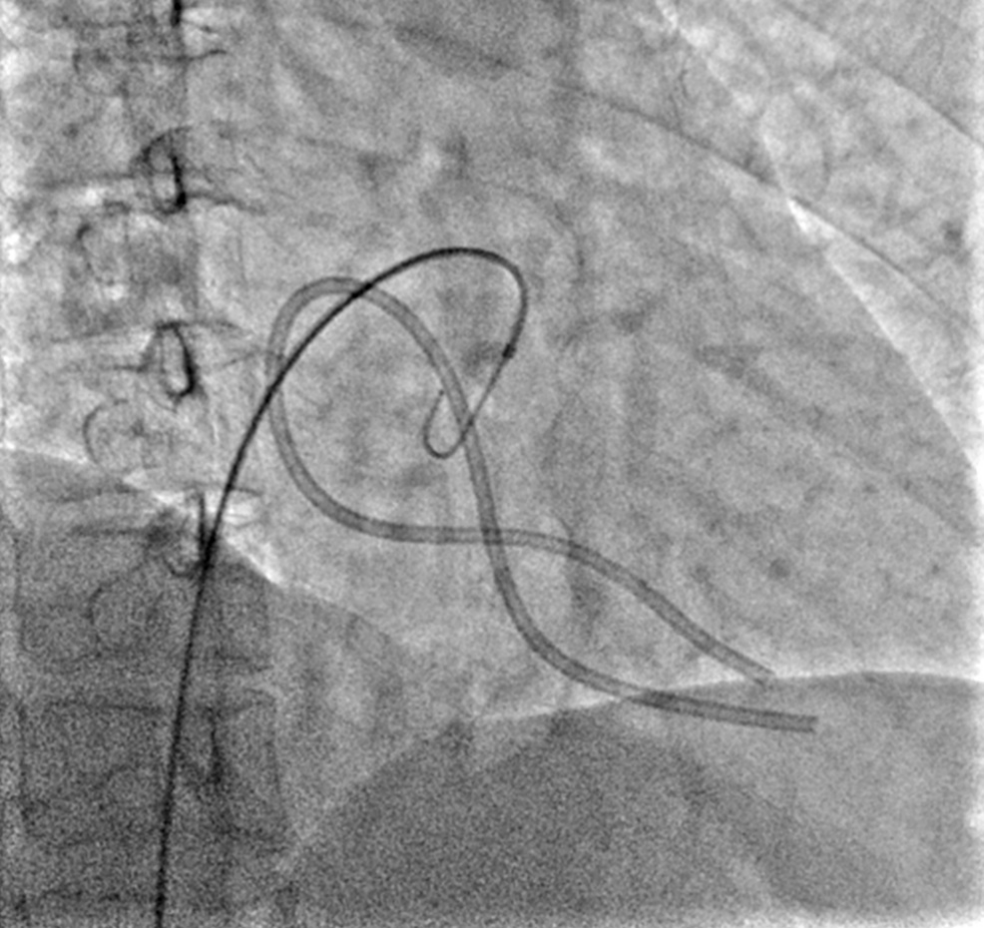
Showing the dislodged chemo port catheter looped in RA with tips in RV and crossing of catheter loop with 5 Fr JR catheter over the 0.035-inch wire. Fr: French gauge; JR: Judkins Right; RA: right atrium; RV: right ventricle

**Figure 3:**
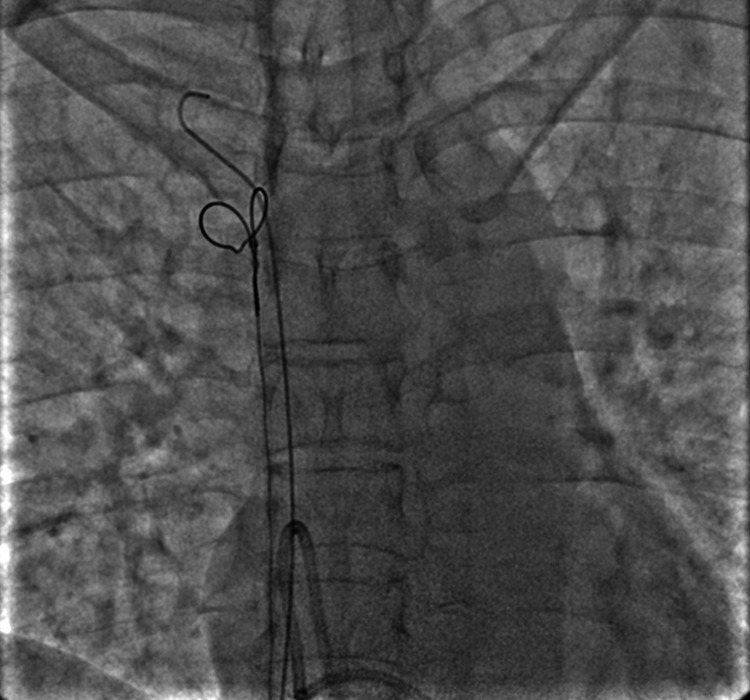
Showing the Amplatz gooseneck snare forming a loop over the 0.035-inch wire.

**Figure 4:**
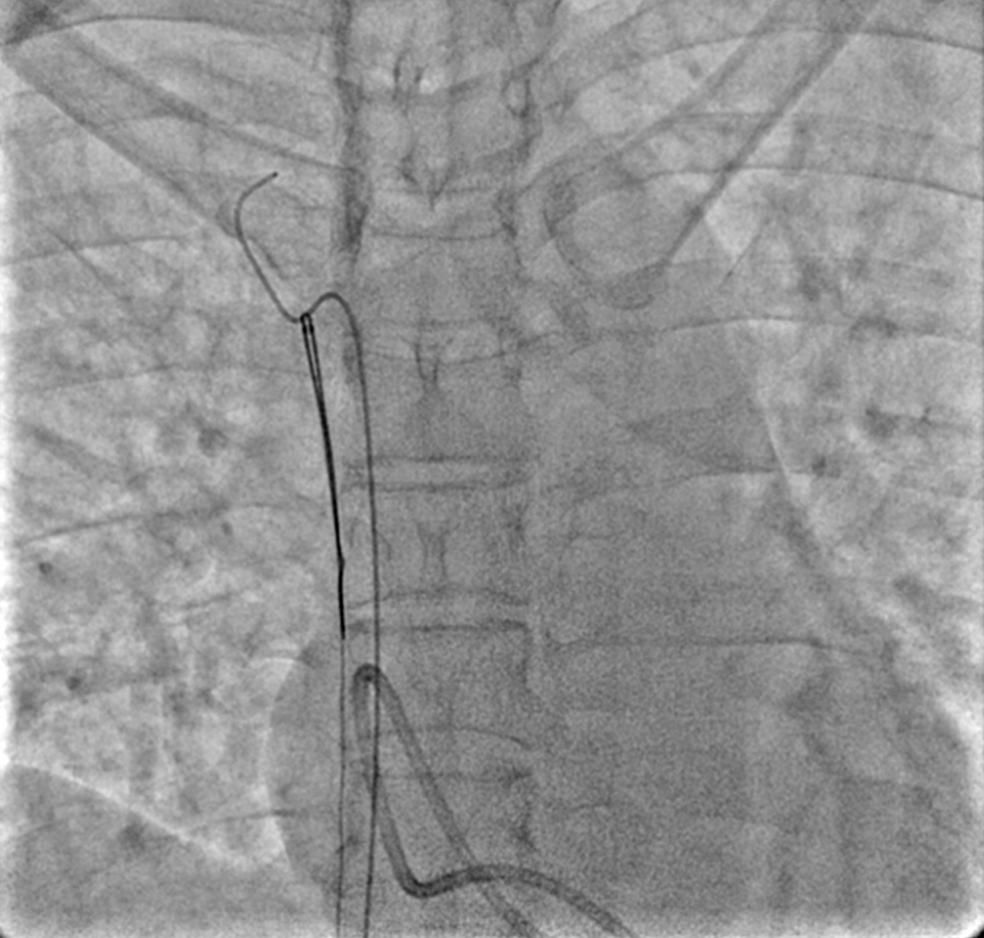
Showing the Amplatz gooseneck snare being closed to catch the wire.

**Figure 5:**
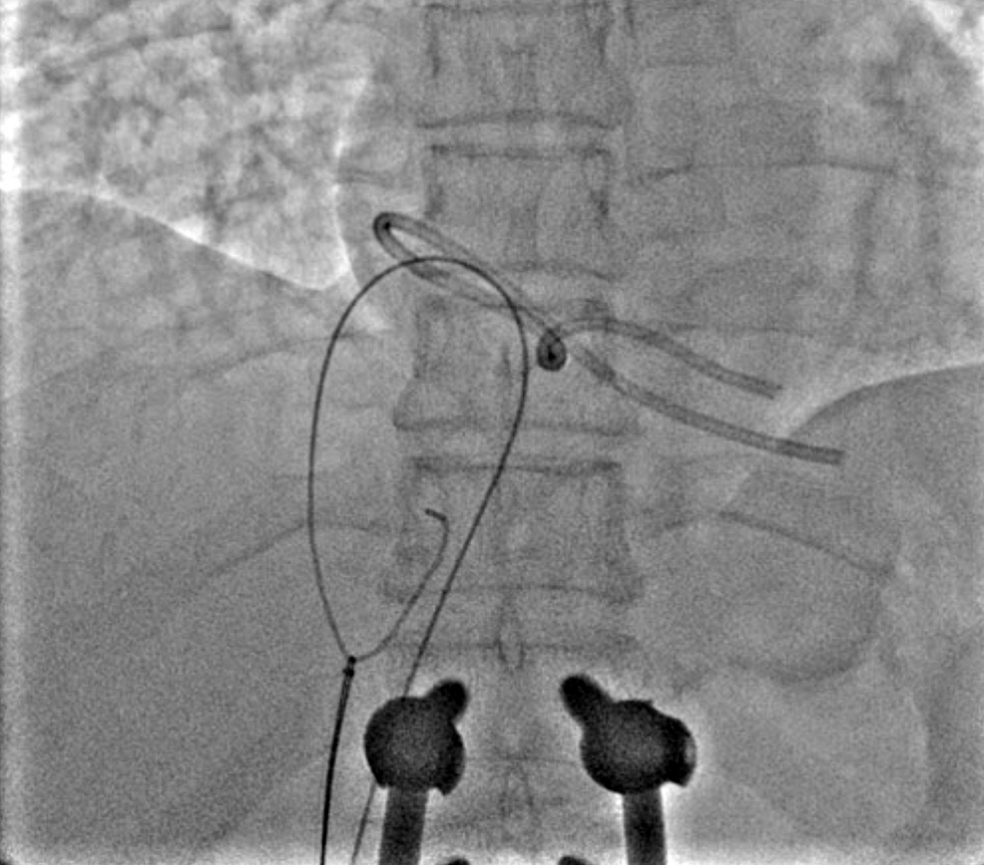
Showing wire and snare loop being pulled down to reposition the catheter.

**Figure 6:**
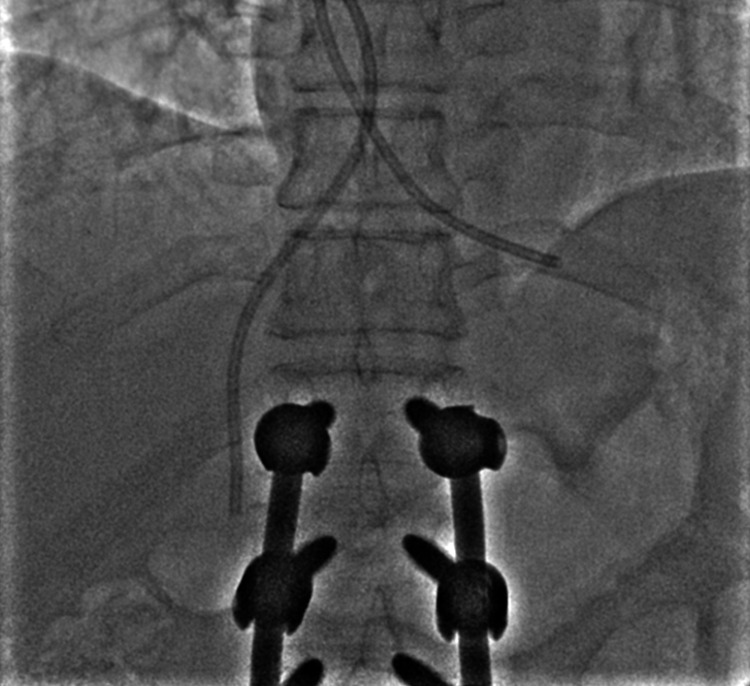
Showing the repositioned catheter with an accessible tip in the IVC. IVC: inferior vena cava

**Figure 7:**
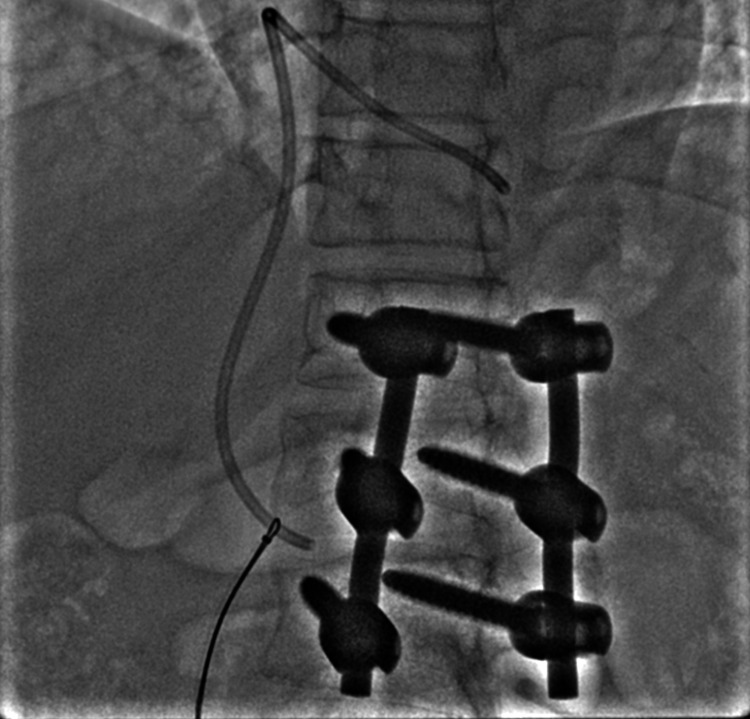
Showing snare being used to catch the free end of the catheter in the IVC. IVC: inferior vena cava

**Figure 8:**
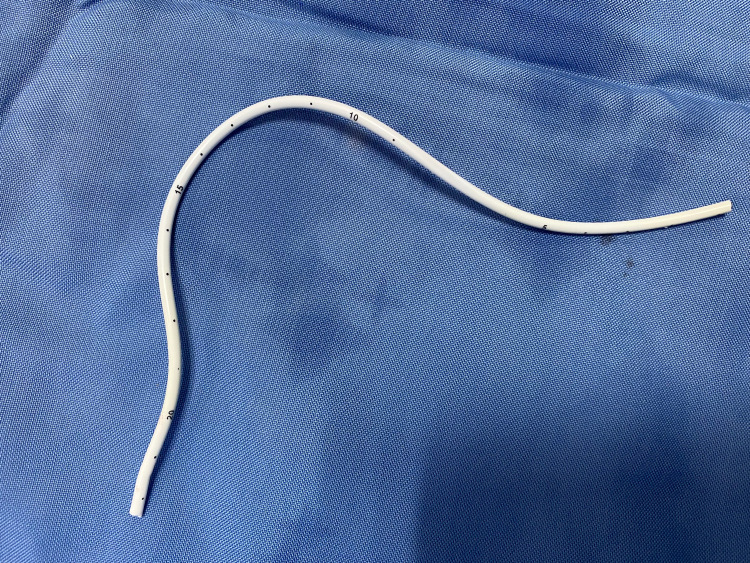
Showing the fractured segment of the chemo port catheter after removal.

The JR catheter was used to cross the loop with the wire and was then withdrawn (Figure 2). Then, the wire was advanced into the SVC. After checking that the wire was within the catheter loop, an Amplatz Goose-Neck 35 mm diameter snare (Medtronic Plc., Dublin, Ireland) was introduced. The snare was used to catch the free end of the guidewire from the SVC and the assembly was pulled down (Figures 3-5). This led to the unlooping of the fragment and its free end was now in the IVC (Figure 6). After removing the wire, the snare was withdrawn into the IVC and readvanced to catch the distal tip of the dislodged fragment in the IVC, and the assembly was successfully pulled out (Figure 7). The cine runs of the entire procedure are shown in Video 1. The length of the dislodged segment was 11 cm (Figure 8). The total fluoroscopy time was three minutes and 40 seconds, and the radiation dose was 81 mGy.

**Video 1:** Showing the cine runs of the entire procedure.

## Discussion

Embolization of fractured fragments of CVPCs has been well described in the literature. However, these are relatively rare occurrences. The most common location of the fracture described in the literature is the anastomotic site between the ports and catheter, likely due to the constant shear stress at that location [6]. Dislodged catheter fragments in the heart may lead to multiple complications, most commonly infective endocarditis, necessitating emergent removal [7]. In a series of 92 patients by Cheng et al. with dislodged CVPCs, the most common location of dislodgement was the SVC-RA junction [6]. In the same series, the least common sites of dislodgement were the RA to the coronary sinus and main pulmonary artery. Similar to the present case, the presence of the catheter in RA-RV was seen in 10 (10.86%) cases.

Though in most cases direct snaring is feasible, cases like the present one wherein the tips are not easily accessible present a challenge. Cheng et al. have described the use of wire loops, pigtail catheters, and dormia baskets for catheter retrievals in such situations [6]. There have been many reports of using wire loops to pull out or reposition dislodged catheters with inaccessible ends. Haga and Shindo have described a modified loop snare technique wherein, similar to the present case, a wire was looped around the dislodged catheter to reposition it into the IVC and then directly snared out [8]. Another strategy to retrieve catheters with inaccessible ends is the use of pigtail catheters [6,9]. The uniqueness of the present case lies in the fact that the catheter loop was rather shallow and deeply placed in the RV. Direct looping of the wire around the catheter to reposition it was not feasible. Hence, a wire was passed inside the catheter loop and its end was caught with a snare to create a neo-loop to straighten out the dislodged catheter into the IVC.

Percutaneous retrieval of dislodged catheters is safe and effective with a success rate of >90%. Surgical intervention has been resorted to in only rare situations. In the 92-patient series of Cheng et al., there were only two failed procedures [6]. In another series of 25 patients by Wang et al., there were no procedural failures by the percutaneous route [10]. However, in two of 25 patients, snaring failed, and the procedures were completed using grasping and myocardial biopsy forceps. A similar 100% success rate of percutaneous retrieval has also been reported by Li et al. in a 10-patient series [11]. The procedures have been documented to be safe with no major periprocedural complications. However, Wang et al. have reported transient arrhythmias in a few patients, with no major adverse events due to the same [10].

## Conclusions

Anatomical challenges in snaring occur when the dislodged fragments are in relatively inaccessible locations, such as deep in the RV and proximal pulmonary vasculature. In such cases, similar to the present one, improvised techniques using routinely available endovascular hardware can lead to procedural success even in the most difficult of cases.

## References

[1] Patel T, Shah S, Pandya R, Sanghvi K, Fonseca K (2000). Broken guidewire fragment: a simplified retrieval technique. Catheter Cardiovasc Interv.

[2] Kwan TW, Chaudhry M, Huang Y (2013). Approaches for dislodged stent retrieval during transradial percutaneous coronary interventions. Catheter Cardiovasc Interv.

[3] Patel T, Shah S, Pancholy S (2018). Patel’s atlas of complications of coronary interventions: plan-B. Managing Trapped Devices in the Coronary and Peripheral Circulation.

[4] Kock HJ, Pietsch M, Krause U, Wilke H, Eigler FW (1998). Implantable vascular access systems: experience in 1500 patients with totally implanted central venous port systems. World J Surg.

[5] Charvát J, Linke Z, Horáèková M, Prausová J (2006). Implantation of central venous ports with catheter insertion via the right internal jugular vein in oncology patients: single center experience. Support Care Cancer.

[6] Cheng CC, Tsai TN, Yang CC, Han CL (2009). Percutaneous retrieval of dislodged totally implantable central venous access system in 92 cases: experience in a single hospital. Eur J Radiol.

[7] Alizadehasl A, Zohrian F, Abdi S (2020). Echocardiographic and angiographic guided removal of a fractured central venous access port using snare loop. Multidisc Cardiovasc Ann.

[8] Haga M, Shindo S (2020). A modified loop snare technique for the retrieval of a dislodged central venous catheter. Radiol Case Rep.

[9] Mori K, Somagawa C, Kagaya S, Sakai M, Homma S, Nakajima T (2021). "Pigtail through snare" technique: an easy and fast way to retrieve a catheter fragment with inaccessible ends. CVIR Endovasc.

[10] Wang PC, Liang HL, Wu TH (2009). Percutaneous retrieval of dislodged central venous port catheter: experience of 25 patients in a single institute. Acta Radiol.

[11] Li Y, Chen J, Li Z, Lu H, Ren K, Ren J, Han X (2020). Successful percutaneous transvenous retrieval of intravascular fractured port catheter: a single center experience. J Cardiothorac Surg.

